# "Overtime Hours Effect" on Emergency Surgery of Acute Type A Aortic Dissection

**DOI:** 10.21470/1678-9741-2018-0350

**Published:** 2019

**Authors:** Orhan Gokalp, Levent Yilik, Yuksel Besir, Hasan Iner, Nihan KarakasYesilkaya, Erturk Karaagac, Yasar Gokkurt, Sahin Iscan, Ali Gurbuz

**Affiliations:** 1 Department of Cardiovascular Surgery, Izmir Katip Celebi University, Faculty of Medicine, Izmir, Turkey.; 2 Department of Cardiovascular Surgery, Izmir Katip Celebi University, Ataturk Education and Research Hospital, Izmir, Turkey.

**Keywords:** Reoperation, Aneurysm, Dissection, Aorta, Pulmonary Disease, Chronic Obstructive, Acute Diseases, Morbity

## Abstract

**Objective:**

Treatment of acute diseases of the aorta is still associated with high mortality and morbidity. It is believed that interventions for these diseases on overtime hours (night shifts or weekend shifts) may increase mortality. In this study, we investigated the effect of performing acute type A aortic dissection surgery on overtime hours in terms of postoperative outcomes.

**Methods:**

206 patients who underwent emergency surgery for acute type A aortic dissection were retrospectively evaluated. Two groups were constituted: patients operated on daytime working hours (n=61), and patients operated on overtime hours (n=145), respectively.

**Results:**

Chronic obstructive pulmonary disease and repeat surgery were higher in group 1. There was no statistically significant difference between the two groups in terms of operative and postoperative results. Mortality rates and postoperative neurological complications in group 1 were 9.8% and 13.1%, respectively. In group 2, these rates were 13.8% and 12.4%, respectively (*P*=0.485 - *P*=0.890). Multivariate analysis identified that cross-clamp time, amount of postoperative drainage, preoperative loss of consciousness and postoperative neurological complications are the independent predictors of mortality.

**Conclusions:**

As the surgical experience of the clinics improves, treatment of acute type A aortic dissections can be successfully performed both overtime and daytime working hours.

**Table:** 

Abbreviations, acronyms & symbols	
ATAAD	= Acute type A aortic dissection
CC	= Cross-clamp
COPD	= Chronic obstructive pulmonary disease
CPB	= Cardiopulmonary bypass
EJCTS	= European Journal of Cardiothoracic Surgery
EuroSCORE	= European System for Cardiac Operative Risk Evaluation
ICVTS	= Interactive Cardiovascular and Thoracic Surgery
MACCEs	= Major adverse cardiac and cerebrovascular events

## INTRODUCTION

Acute type A aortic dissection (ATAAD) is a catastrophic phenomenon with a high mortality rate, regardless of the concomitant cardiac disease. The mortality rate varies between 7% and 30%, even in operated patients^[1,5]^. Variables that affect the outcome of this pathology are rarely investigated. In the literature, it has been indicated that several factors affect mortality and morbidity after ATAAD surgery. These factors include history of aortic valve replacement, migrating chest pain, hypotension and/or shock, cardiac tamponade, extremity ischemia, duration of cardiopulmonary bypass, and chronic renal failure. In investigations on the effect of surgical technique, the efficacy of antegrade, retrograde, or selective cerebral perfusion following circulatory arrest remains a controversial topic^[1,3]^.

On the other hand, performing operations on overtime hours has been indicated among the factors that affect surgical outcomes in several studies^[6,9]^. Studies evaluating this controversial issue suggested intermittent health service, presence of fewer and less experienced personnel in hospitals during overtime hours as an explanation for this situation^[9,10]^. This situation is commonly described as ‘weekend effect’ in the literature^[7,10]^. However, no study has investigated the effect of this situation on the outcome of ATAAD. In this study, we aimed to compare the outcome of ATAAD surgeries performed during working hours and overtime hours.

## METHODS

This study retrospectively evaluated 206 patients who underwent emergency surgery for ATAAD between January 2000 and May 2018. Local ethics committee approval was obtained for the study (05/07/2017-147). Patients who underwent surgery on daytime working hours constituted group 1 (n=61) and those who underwent surgery on overtime hours constituted group 2 (n=145). Overtime working hours were accepted from 5 p.m. to 8 p.m. from Monday to Friday and 24 hours from Saturday to Sunday. We compared the groups in terms of operative and postoperative outcomes. We also searched for predictors of mortality and postoperative neurological complications.

Hospital records were used to retrospectively evaluate pre- and postoperative clinical variables. Age, sex, comorbid factors, redo cardiac surgery, ascending aorta diameter at admission, body surface area, EuroSCORE, hemoglobin, white blood cell count, preoperative malperfusion signs, and ejection fraction were recorded as preoperative parameters. History of coronary ischemia, limb ischemia, cerebrovascular event, acute renal failure, and signs of ileus were accepted as organ malperfusion. Operating time, cardiopulmonary bypass (CPB) time, cross-clamp (CC) time, intervention to aortic root or aortic arch were evaluated as operative variables. Postoperative variables included especially mortality and neurological complications in addition to other parameters such as weaning time, intensive care unit stay, hospital stay, development of renal failure, blood transfusion, and revision surgery for postoperative bleeding.

Contrasted-enhanced thoracoabdominal computed tomography and transthoracic echocardiography were performed preoperatively in all patients. The patients underwent emergency surgery. All operations were performed by different surgeons with similar surgical techniques. To perform antegrade cerebral perfusion, all patients underwent right axillary arterial cannulation. All the distal anastomoses of the grafts interposed to the aorta were performed under total circulatory arrest with open technique. Weaning period longer than 24 hours after operation was regarded as prolonged intubation. Patients who underwent elective surgery due to subacute or chronic type A aortic dissection were excluded from the study.

### Endpoints

Primary endpoints compared between the groups femoral cannulation and axillar cannulation were defined following the European Journal of Cardiothoracic Surgery (EJCTS) and Interactive Cardiovascular and Thoracic Surgery (ICVTS) Statistical and Data Reporting Guidelines^[11]^: operative mortality, defined as all-cause mortality at 30 days; occurrence of postoperative neurological injury at 30 days; major adverse cardiac and cerebrovascular events (MACCEs), a composite endpoint of all-cause mortality, myocardial infarction, need for emergent cardiac surgery or percutaneous intervention and stroke up to 30 days postoperatively.

### Statistical Method

Data were presented as mean, standard deviation, median, minimum, maximum, frequency and percentage. The distribution of variables was evaluated with Kolmogorov-Smirnov test. To analyze the continuous variables, we used independent samples *t*-test or Mann-Whitney U test and to analyze the categorical variables, we used chi-square test or Fisher’s exact test. Univariate and multivariate logistic regression was used to analyze the impact of variables on postoperative outcomes. A *P*-value lower than 0.05 was considered statistically significant. We used IBM SPSS 22.0 for the statistical analysis.

## RESULTS

Chronic obstructive pulmonary disease (COPD) and repeat surgery were higher in group 1. Among patients with COPD, it was significantly more common in patients in group 1 (47.5-23.4%, *P*=0.001). Similarly, repeat surgery rate was higher in group 1 (29.5-16.6%, *P*=0.035). Other preoperative variables were similar in both groups (Table 1).

**Table 1.** Preoperative data.

		Standard working hours (group 1) n=61		Overtime hours (group 2) n=145		*P*
		Mean±sd/n-%	Med	Mean±sd/n-%	Med	
Age		54.7±12.5	56.0	57.5±11.8	58.0	0.218^m^
Gender	Female	27 44.3%		64 44.1%		0.987^X2^
	Male	34 55.7%		81 55.9%		
DM		18 29.5%		30 20.7%		0.172^X2^
COPD		29 47.5%		34 23.4%		0.001^X2^
Smoking		36 59.0%		78 53.8%		0.491^X2^
CRF		2 3.3%		4 2.8%		1.000^X2^
HT		50 82.0%		101 69.7%		0.068^X2^
Marfan		4 6.6%		14 9.7%		0.472^X2^
BSA		1.8±0.1	1.8	1.8±0.1	1.8	0.153^m^
Use of antiaggregants		12 19.7%		29 20%		0.957^X2^
Repeat surgery		18 29.5%		24 16.6%		0.035^X2^
Ascending aorta diameter		55.6±9.7	53.0	53.6±10.3	52.0	0.210^m^
EuroSCORE		5.4±1.2	5.0	5.3±1.5	5.0	0.548^m^
Malperfusion		6 9.8%		14 9.7%		0.968^X2^
Loss of consciousness		6 9.8%		17 11.7%		0.694^X2^
Shock		4 6.6%		11 7.6%		0.795^X2^
CPR		1 1.6%		3 2.1%		1.000^X2^
Tamponade or effusion		9 14.8%		22 15.2%		0.939^X2^
Hemoglobin (g/dL)		11.9±1.7	11.8	11.5±1.7	11.5	0.266^m^
WBC >11,000 (K/uL)		29 47.5%		70 48.3%		0.923^X2^
EF <50		17 27.9%		42 29.0%		0.874^X2^

DM=diabetes mellitus, COPD=chronic obstructive pulmonary disease, CRF=chronic renal failure, HT=hypertension, BSA=body surface area, CPR=cardiopulmonary resuscitation, WBC=white blood cells, EF=ejection fraction, tt test.  m: Mann-Whitney u test. X²: Chi-square test (Fisher’s test).

Operative variables were not significantly different between the groups. Namely, similar operations in similar durations were performed (Table 2).

**Table 2.** Perioperative data.

	Standard working hours (group 1) n=61		Overtime hours (group 2) n=145		*P*
	Mean±sd/n-%	Med	Mean±sd/n-%	Med	
Cross-clamp time (min)	121.9±36.1	116.0	123.2±37.7	120.0	0.835^m^
CPB time (min)	181.0±59.7	181.0	184.7±55.4	176.0	0.614^m^
Operating time (min)	340.0±93.1	322.0	342.9±81.1	330.0	0.840^m^
Root intervention	23 37.7%		38 26.2%		0.099^X2^
Hemiarch intervention	26 42.6%		48 33.1%		0.194^X2^
Total arch intervention	11 18.0%		32 22.1%		0.515^X2^
Only ascending aortic intervention	24 39.3%		65 44.8%		0.468^X2^
Weaning time (H)	25.8±19.3	22.0	30.9±25.2	23.5	0.201^m^
ICU time (days)	5.6±4.7	4.0	6.5±13.9	4.0	0.293^m^
Hospital discharge time (days)	11.9±7.0	9.0	13.1±13.8	10.0	0.527^m^
Postoperative drainage (ml)	874±413	850	903±525	800	0.827^m^
Blood product (IU)	3.5±2.5	3.0	4.0±3.2	3.0	0.331^m^
POAKI	9 14.8%		25 17.2%		0.661^X2^
Reoperation for bleeding	20 32.8%		47 32.4%		0.958^X2^
Prolonged intubation	27 44.3%		75 51.7%		0.328^X2^
Neurological complication	8 13.1%		18 12.4%		0.890^X2^
Mortality	6 9.8%		20 13.8%		0.485^X2^

CPB=cardiopulmonary bypass; ICU=intensive care unit, POAKI=postoperative acute kidney injury. m: Mann-Whitney U test; X^2^ Chi-square test (Fisher’s test).

The findings that constitute the main subject of our study emerged in postoperative data. First, early mortality had no statistical difference between the two groups (6, 9.8% *vs*. 20, 13.8%, *P*=0.485). Similarly, two groups were similar in terms of weaning period, intensive care unit stay, hospital stay, acute renal failure requiring dialysis, the amount of blood transfusion and revision surgery for postoperative bleeding. While postoperative neurological complication rate was 13.1% (n=8) in patients operated on daytime working hours, it was 12.4% (n=18) in patients operated on overtime hours shifts (*P*=0.890).

Another aim of this study was to determine the mortality predictors in all patients regardless of the operating time. In the univariate model, CPB time, CC time, operation time, amount of postoperative drainage, intensive care unit stay, use of blood products, malperfusion, loss of consciousness preoperatively, revision surgery for postoperative bleeding, acute renal failure requiring dialysis, prolonged intubation, and neurological complication predicted early mortality (P˂0.05). In the multivariate model, CC time, amount of postoperative drainage, loss of consciousness preoperatively and neurological complication were independent predictors of early mortality (*P*<0.05) (Table 3).

**Table 3.** Predictors of mortality.

	Univariate analysys			Multivariate analysys		
	OR	95% CI	*P*	OR	95% CI	*P*
Cardiopulmonary bypass time	1.02	1.01-1.02	*0.000*			
Cross-clamp time	1.03	1.02-1.05	*0.000*	1.03	1.01-1.05	*0.001*
Operating time	1.01	1.00-1.01	*0.000*			
Drainage	1.00	1.00-1.00	*0.000*	1.00	1.00-1.00	*0.033*
Intensive care unit time	1.01	0.99-1.04	*0.243*			
Blood products	1.35	1.17-1.54	*0.000*			
Malperfusion	3.55	1.22-10.29	*0.019*			
Loss of consciousness	39.54	13.25-117.9	*0.000*	26.8	6.06-118.61	*0.000*
Reoperation for bleeding	4.91	2.05-11.7	*0.000*			
Acute renal failure requiring dialysis	11.55	4.64-28.7	*0.000*			
Prolonged intubation	6.87	2.27-20.7	*0.001*			
Neurological complication	20.95	7.79-56.3	*0.000*	11.9	2.76-51.37	*0.001*

In the univariate analysis of the postoperative neurological complications predictors, smoking, CPB time, CC time, operating time, amount of postoperative drainage, intensive care unit stay, use of blood products, hospital stay time, WBC >11,000 (K/uL), loss of consciousness preoperatively, reoperation for bleeding, acute renal failure requiring dialysis and prolonged intubation were significant (*P*<0.05). In the multivariate model, smoking, CPB time, WBC >11,000 (K/uL) and acute renal failure requiring dialysis were the independent predictors of neurological complication (*P*<0.05) (Table 4).

**Table 4.** Neurological complication predictors.

	Univariate analysis			Multivariate analysis		
	OR	95% CI	*P*	OR	95% CI	*P*
Smoking	3.05	1.17-7.94	*0.023*	6.208	1.747-22.06	0.005
Cardiopulmonary bypass time	1.01	1.00-1.01	* 0.001*	1.01	1.01-1.02	0.030
Cross-clamp time	1.02	1.01-1.03	*0.000*			
Operating time	1.00	1.00-1.01	*0.015*			
Drainage	1.00	1.00-1.00	*0.000*			
Intensive care unit time	1.02	0.99-1.0 5	*0.116*			
Blood products	1.23	1.09-1.38	*0.001*			
Hospital discharge time	1.01	0.98-1.03	*0.319*			
WBC >11,000 (K/Ul)	2.75	1.13-6.65	*0.025*	3.77	1.05-13.50	0.041
Loss of consciousness	8.02	3.04-21.2	*0.000*			
Reoperation for bleeding	4.91	2.05-11.73	*0.000*			
Acute renal failure requiring dialysis	14.4	5.69-36.42	*0.000*	16.13	5.17-50.33	0.000
Prolonged intubation	5.13	1.85-14.21	*0.002*			

## DISCUSSION

The main purpose of this study is to evaluate the effect of overtime hours or weekend hours on ATAAD surgery results. We also aimed to investigate predictors of mortality and neurologic complications as one of the most debatable points about this subject.

ATAAD is a catastrophic condition which has high mortality and morbidity, even for patients diagnosed promptly and underwent emergency surgery. Therefore, these patients should be operated by the most experienced team in a cardiovascular department. However, it is a fact that experienced teams are not always available on overtime hours. This suggests that ATAAD operations performed on overtime hours may have a worse outcome. In fact, this hypothesis has also been proposed in other diseases requiring urgent intervention and investigated accordingly. ‘Weekend effect’ was used to describe this situation^[9,10]^. In general, this phenomenon is explained by intermittent health services and the presence of fewer and less experienced personnel in hospitals during overtime hours^[7,10]^ In a study investigating the impact of overtime hours effect on the management of ruptured abdominal aortic aneurysms, Groves et al.^[12]^ stated: “The necessity of prompt action in the care of ruptured aortic aneurysms makes it an excellent model for evaluation of what is referred to as the weekend effect". Groves et al.^[12]^ found that mortality rate is 32% higher in patients admitted for ruptured aortic aneurysms on overtime hours compared to those applied in daytime working hours. Although they could not explain this finding completely, they suggested delaying the surgeries of patients admitted on weekends to perform surgery in more favorable conditions might have increased the mortality. They supported their idea by demonstrating increased use of blood products in patients admitted on weekends due to delays. In a similar study, Gallerani et al. investigated the weekend effect in 4,461 patients with acute ruptured or dissected aortic aneurysm^[13]^. In their study, the mortality rate was 43.4% in patients admitted on weekends and 36.9% in patients admitted on weekdays. Accordingly, they concluded that applying on weekends is a factor that increases mortality in these conditions. Similar to other studies, they also explained their findings with the presence of fewer or less experienced personnel in the hospitals on weekends. Bell et al. also identified weekend admissions as a predictor of mortality in patients with ruptured abdominal aortic aneurysm^[8]^. In these studies, the impact of weekend effect on the management of these conditions was investigated. On the other hand, we specifically investigated the effect of this phenomenon on surgical outcomes. As patients with ATAAD should be treated urgently and unlike patients in the study of Gallerani et al.^[13]^, it is not possible to delay their operation. Therefore, we investigated more specifically the impact of surgical teams. According to our results, there is no overtime hours effect in terms of postoperative mortality and neurological complication. Aside from this, the lack of differences regarding other operative and early postoperative data indicates that overtime hours effect lost its importance in these conditions. In addition, it is critical to note that all patients in both groups were operated by different surgical teams with similar surgical skills and experiences and with the different surgical technicians.

Some studies have investigated the variables that affect the outcomes of ATAAD. Despite different results from previous studies, factors affecting mortality can be predicted. Macrina et al.^[3]^ evaluated 208 patients operated for ATAAD. They identified age, preoperative shock, intubation and previous cardiac surgery as predictors of mortality. Among operative variables, CC time and CPB time and among postoperative variables, renal complication, need for dialysis and neurological complication were identified as predictors of mortality. ‘The International Registry of Acute Aortic Dissection’ studies highlighted the importance of patient selection in surgical outcomes^[2,14]^. In these studies, cardiac tamponade, shock, congestive cardiac failure, cerebrovascular event, stroke, coma, acute myocardial or mesenteric ischemia and acute renal failure during surgery have been identified as mortality predictors. Grimm et al.^[15]^ assigned 101 patients with ATAAD into two groups according to preoperative malperfusion. While the most effective predictor of mortality was age in patients without malperfusion, it was intestinal ischemia in patients with malperfusion. Patients with preoperative malperfusion were evaluated in a larger study with 2,137 patients^[16]^. When survival was considered, age, peripheral malperfusion, involvement of supra-aortic branches, coronary malperfusion, spinal malperfusion, a primary entry in the descending aorta, and preoperative comatose state were independent predictors, again with increasing significance. At this point, our results are compatible with the literature. Mortality predictors identified by univariate model such as CPB time, CC time, operating time, amount of postoperative drainage, intensive care unit stay, use of blood products, malperfusion, loss of consciousness preoperatively, revision surgery for postoperative bleeding, acute renal failure requiring dialysis, prolonged intubation and neurological complication are similar to previous studies. However, CC time, amount of postoperative drainage, loss of consciousness preoperatively and neurological complication were the independent predictors of mortality according to multivariate analysis.

In our analysis of predictors of postoperative neurological complications, which was the most important independent predictor of mortality, we found the following results. Important factors investigated in previous studies include presence of preoperative cerebral dysfunction, preoperative coma, preoperative myocardial ischemia, intervention to supra-aortic branches during operation and long operation time^[16,17]^. Similar to the literature, we identified the following factors as predictors of postoperative neurological dysfunction: smoking, CPB time, CC time, operating time, amount of postoperative drainage, intensive care unit stay, use of blood products, hospital stay, WBC >11,000 (K/uL), loss of consciousness preoperatively, reoperation for bleeding, acute renal failure requiring dialysis and prolonged intubation.

### Limitations of the Study

Limitations of our study include the monocentric retrospective study design and relatively low number of patients. However, it should be considered that our study is the first one evaluating the relationship between overtime hours effect or weekend effect and ATAAD.

## CONCLUSION

ATAAD surgeries performed on overtime hours by experienced teams have similar outcomes compared to operations performed on daytime working hours. We think the reason for this is the devotion of experienced teams to perform one of the most difficult operations of cardiovascular surgery regardless of time. However, further prospective randomized controlled studies are needed to investigate the impact of the weekend effect in ATAAD.

**Table:** 

Author's roles & responsibilities	
OG	Substantial contributions to the conception or design of the work; or the acquisition, analysis, or interpretation of data for the work; drafting the work or revising it critically for important intellectual content; final approval of the version to be published
LY	Substantial contributions to the conception or design of the work; or the acquisition, analysis, or interpretation of data for the work; drafting the work or revising it critically for important intellectual content; final approval of the version to be published
YB	Acquisition, analysis, or interpretation of data for the work; final approval of the version to be published
HI	Acquisition, analysis, or interpretation of data for the work; final approval of the version to be published
NKY	Acquisition, analysis, or interpretation of data for the work; final approval of the version to be published
EK	Acquisition, analysis, or interpretation of data for the work; final approval of the version to be published
YG	Acquisition, analysis, or interpretation of data for the work; final approval of the version to be published
SI	Acquisition, analysis, or interpretation of data for the work; final approval of the version to be published
AG	Acquisition, analysis, or interpretation of data for the work; drafting the work or revising it critically for important intellectual content; final approval of the version to be published
